# Phosphorylation of p90RSK is associated with increased response to neoadjuvant chemotherapy in ER-positive breast cancer

**DOI:** 10.1186/1471-2407-12-585

**Published:** 2012-12-10

**Authors:** Hyeong-Gon Moon, Jae Kyo Yi, Hee Sung Kim, Hea Young Lee, Kyung-Min Lee, Minju Yi, Sookyung Ahn, Hee-Chul Shin, Ji-hyun Ju, Incheol Shin, Wonshik Han, Dong-Young Noh

**Affiliations:** 1 Department of Surgery and Cancer Research Institute, Seoul National University College of Medicine, Seoul, Korea; 2 Department of Pathology, Gachon University Gil Hospital, Gachon University of Medicine and Science, Incheon, Korea; 3 Department of Surgery, Chung-Ang University College of Medicine, Seoul, Korea; 4 Department of Life Science, College of Natural Science, Hanyang University, Seoul, Korea

**Keywords:** Breast cancer, P90RSK, Chemotherapy, Predictive marker, ERK, Estrogen receptor

## Abstract

**Background:**

The clinical implication of Ras/Raf/ERK pathway activity in breast cancer tissue and its association with response to chemotherapy is controversial. We aimed to explore the value of p90RSK phosphorylation, a downstram molecule of the pathway, in predicting chemotherapy response in breast cancer.

**Methods:**

The expression of phosphorylated p90RSK (phospho-p90RSK) and chemotherapy response was measured in 11 breast cancer cell lines and 21 breast cancer tissues. The predictive value of phospho-p90RSK was validated in core needle biopsy specimens of 112 locally advanced breast cancer patients who received anthracycline and taxane-based neoadjuvant chemotherapy.

**Results:**

In 11 breast cancer cell lines, the relative expression of phospho-p90RSK was inversely correlated with cell survival after doxorubicin treatment (p = 0.021). Similar association was observed in fresh tissues from 21 breast cancer patients in terms of clinical response. In paraffin-embedded, formalin-fixed tissues from core needle biopsy tissues from 112 patients, positive phospho-p90RSK expression was associated with greater tumor shrinkage and smaller post-chemotherapy tumor size. The association between phospho-p90RSK expression and chemotherapy response was more evident in estrogen receptor(ER)-positive tumors. The expression of phosphor-p90RSK did not show a significant relationship with the incidence of pCR. P90RSK silencing using siRNA did not affect the cancer cell’s response to doxorubicin, and the expression of phospho-p90RSK was highly correlated with other Ras/Raf/ERK pathway activation.

**Conclusion:**

Our results suggest that phospho-p90RSK expression, which reflects the tumor’s Ras/Raf/ERK/p90RSK pathway activation can be a potential predictive marker for chemotherapy response in ER-positive breast cancer which needs further independent validation.

## Background

Breast cancer is the most common solid cancer in women worldwide. Recent improvement in breast cancer survival is largely due to increased early detection and development of effective systemic chemotherapeutic agents [1]. Currently, a significant proportion of breast cancer patients received adjuvant systemic chemotherapy since meta-analysis results have shown that adjuvant systemic chemotherapy is beneficial regardless of the age and estrogen receptor (ER) expression [2]. Recent efforts are focused in developing molecular markers which would identify a subset of patients in whom the benefit of the cytotoxic chemotherapy is minimal and can be omitted. Various multi-gene signatures were proposed to predict the clinical benefit of chemotherapy in breast cancer, however, only the 21-recurrence score (Oncotype Dx) is currently approved for this purpose [3].

Ras/Raf/ERK signaling pathway has been shown to be involved in intrinsic resistance to endocrine therapy in breast cancer while its role in developing resistance to cytotoxic chemotherapy is controversial [4,5]. Small et al. [6] have previously shown that treatment with anthracycline in various breast cancer cell lines resulted in activation of ERK1/2 pathway and increased phosphorylation of its downstream molecule 90 kDa ribosomal S6 kinase (p90RSK) in a time-dependent manner. Furthermore, the mitogen-activated protein kinase (MAPK) phosphatase which downregulate p90RSK can modulate the chemotherapy-sensitivity in various cancer cell lines [7]. Recent studies have further suggested the importance of p90RSK dysregulation in breast cancer development and progression [8,9]. However, the clear role of this Ras/Raf/ERK/p90RSK pathway in modulating chemotherapy responsiveness is often difficult to estimate in ER-positive breast cancer who receive both endocrine and cytotoxic systemic therapies since the pathway clearly participate in developing endocrine resistance [10].

In this study, we evaluated the predictive value of the phosphorylated p90RSK expression in terms of chemotherapy responsiveness in various breast cancer cell lines. The clinical value of phospho-p90RSK was further tested in locally advanced breast cancer patients who underwent neoadjuvant systemic chemotherapy which is a valuable platform to test the in vivo chemotherapy-sensitivity [11].

## Methods

### Patients and treatments

The Seoul National University Breast Care Center database includes clinicopathologic informations of the breast cancer patients treated at Seoul National University Hospital since 1990 [12]. From the database, we identified patients with locally advanced breast cancer who underwent doxorubicin and taxane-based neoadjuvant chemotherapy. For immunohistochemistry against phospho-p90RSK, we were able to identify 112 patients who had locally advanced breast cancer, underwent doxorubicin and taxane-based neoadjuvant chemotherapy between Jan 2010 and Dec 2011, did not receive HER2-directed targeted therapies, and had available pre-chemotherapy and post-chemotherapy magnetic resonance imaging for response determination. For western blotting against phospho-p90RSK, patients whose fresh frozen tissues were available were selected from database. Tissues were obtained during diagnostic ultrasonography-guided core needle biopsy procedures and stored at -80C. Informed consent was obtained from all patients and the study was approved by the institutional review board of Seoul National University Hospital. All experiments and analyses were done in accordance with the Declaration of Helsinki.

Our neoadjuvant chemotherapy regimens for locally advanced breast cancer patients were previously described [13]. Briefly, patients received docetaxel (75 mg/m^2^ or 60 mg/m^2^) and doxorubicin (60 mg/m^2^ or 50 mg/m^2^) via intravenous infusion every three weeks with granulocyte colony stimulating factor as primary prophylaxis, or doxorubicin (60 mg/m^2^ or 50 mg/m^2^) and cyclophosphamide (600 mg/m^2^) followed by docetaxel (75 mg/m^2^ or 60 mg/m^2^).

### Cell culture and chemoetherapeutic agent

MCF10A, MCF7, MDA-MB-231, MDA-MB-436 and MDA-MB-453 cell lines were obtained from the American Type Culture Collection (ATCC), MDA-MB-468, ZR75-1, BT474, Hs578T and T47D cell lines were obtained from Korea Cell Bank (KCB). MCF10A Cell line was grown in DMEM/F12 (Gibco) media with 5% horse serum (Invitrogen), 1% penicillin/streptomycin (Gibco), 0.5 μg/ml hydrocortisone (Sigma), 100 ng/ml cholera toxin (sigma), 10 μg/ml insulin (Sigma), and 20 ng/ml recombinant human EGF (Invitrogen). MDA-MB-231, MDA-MB-436, MDA-MB-453, MDA-MB-468 and Hs578T cell lines were cultured in DMEM (Gibco) with 10% fetal bovine serum (FBS) and 1% penicillin/streptomycin. All other cell lines were grown in RPMI 1640 with 10% FBS and 1% penicillin/streptomycin. Doxorubicin was purchased from Sigma-Aldrich. The expression status of ER and HER2 in various breast cancer cell lines was determined by the works of Subik et al. [14] and Neve et al. [15].

### Sphere culture and cell growth

Spheres were generated from single cells of lines MCF7 and MDA-MB-231 seeded at 103 cells in 10 mm low attachment plates (Falcon) and cultured in serum-free DMEM (Dulbecco’s Modified Eagles Medium):F12 = 3:1 medium supplemented with 20 ng/mL epidermal growth factor (EGF; Invitrogen), 20 ng/mL basic fibroblast growth factor (bFGF; Millipore), 10 ng/mL leukemia inhibitory factor (LIF, Millipore), B27 supplement (Invitrogen) and antibiotic-antimycotic (Invitrogen). Cells were grown under these conditions as nonadherent spherical clusters. The medium was replenished every 3 ~ 4 days, and cells were obtained after 1 week.

Cells were seeded and grown in the optical density of cells in 100 mm culture dishes and 10 nM doxorubicin was added 24 hours later. An equivalent volume of sterile water (vehicle) was added as a control. At designated times (72 hours) the cells were harvested, stained with trypan blue (Invitrogen), and counted with a hemocytometer. Three to five independent assays were performed for each of the experiments.

### Tissue protein extaction and western blot analysis

To extract total protein, all tissues were weighed and placed in homogenization buffer at a ratio of 100 mg tissue per 0.25 mL using total protein extraction kit (Chemicon International), according to the manufacturer’s instructions. The homogenates were rotated and centrifuged for 20 minutes at 4 oC. Following centrifugation, collected the supernatant and total protein concentration was determined with the Bradford assay using a Bio-Rad Protein Assay kit (Bio-Rad Laboratories), according to the manufacturer’s instructions. Cells were washed twice with PBS, and total cell lysates prepared in lysis buffer using total protein extraction kit (Chemicon), equal amounts of cells or tissue lysates were separated by SDS-PAGE gel.

The antibodies against total p90RSK (32D7), Phospho-p90RSK (Thr359/Ser363), Phospho-Bad (Ser112, 40A9), p44/42 MAPK (137 F5) and Phospho-p44/42 MAPK (Thr202/Thr204, D13.14.4E) were purchased from Cell Signaling Technology, whereas alpha-tubulin (B-7) and BAD (C-7) were purchased from Santa Cruz Biotechnology. Primary antibodies were detected using horseradish peroxidase–linked anti-mouse anti-rabbit conjugates as appropriate (DAKO), and visualized using the enhanced chemiluminescence detection system (Amersham Biosciences). Protein expression levels were quantified using the software ImageJ to detect intensity of the protein bands.

### Immunohistochemical staining

The paraffin-embedded formlin-fixed core needle biopsy tissues from the above-mentioned 112 patients before the initiation of neoadjuvant chemotherapy were collected for phospho-p90RSK immunohistochemical staining. Serial sections from formalin-fixed, paraffin-embedded (FFPE) blocks were applied to 3-aminopropyltriethoxysilane-coated slides. Deparaffinization and rehydration were performed using xylene and alcohol. The slides were pretreated in a microwave oven for antigen retrieval. Sections were incubated for 30 min at room temperature with antibodies against phospho-p90RSK (1:50 dilution, Ab #9344, Cell Signaling, MA). To block endogenous peroxidase activity, treatment with blocking reagent (DAKO, Glostrup, Denmark) for 5 min was carried out before incubation with primary antibody for 30 min at 25°C. Enzyme-conjugated polymer (DAKO) and diaminobenzidine (DAKO) were used as a visualization system and chromogen, respectively. The phospho-p90RSK expression was measured by evaluating both intensity and area. Most normal duct epithelial cells showed weak or strong positive staining in variable% of cells. Stromal cells were negative. Tumors showing weak nuclearcytoplasmic staining of phospho-pRSK in more than 50% cells or stronge nuclearcytoplasmic staining in more than 20% of cells were considered to be positive for phospho-p90RSK expression.

### Definition of phenotype and response to neoadjuvant chemotherapy

ER, PR, and HER2 expression patterns were evaluated with the standard avidin–biotin complex immunohistochemical staining method, as described previously [16]. The ER and PR results were interpreted as positive when more than 10% of tumor cells showed positive nuclear staining. Tumors with indeterminate HER2 immunohistochemistry results were further evaluated using FISH.

Magnetic resonance imaging is the most accurate method of assessing the residual tumor extent in patients who undergo neoadjuvant chemotherapy when compared to mammography or ultrasonography [17]. Therefore, we used the pre-chemotherapy and the post-chemotherapy magnetic resonance imaging to determine the degree of tumor response. Our protocol for breast cancer magnetic resonance imaging was previously reported [17]. Pathologic complete response (pCR) was defined as the absence of residual invasive tumor cells at the primary tumor site as defined by the NSABP [18].

### Statistic analysis

The chi-square test and Student’s t test were used to compare clinicopathologic variables between groups. Mann–Whitney test was used to determine the difference in phosphor-p90RKS expression between 10 chemo-sensitive tumors and 10 chemo-resistant tumors measured by western blotting. Pearson’s correlation analysis was used to determine the relationship between phosphor-p90RSK expression and other Raf/MEK/ERK pathway molecules. All statistical analyses were performed using IBM SPSS Statistic software version 19 (IBM, Armonk, New York).

## Results

**Figure 1:**
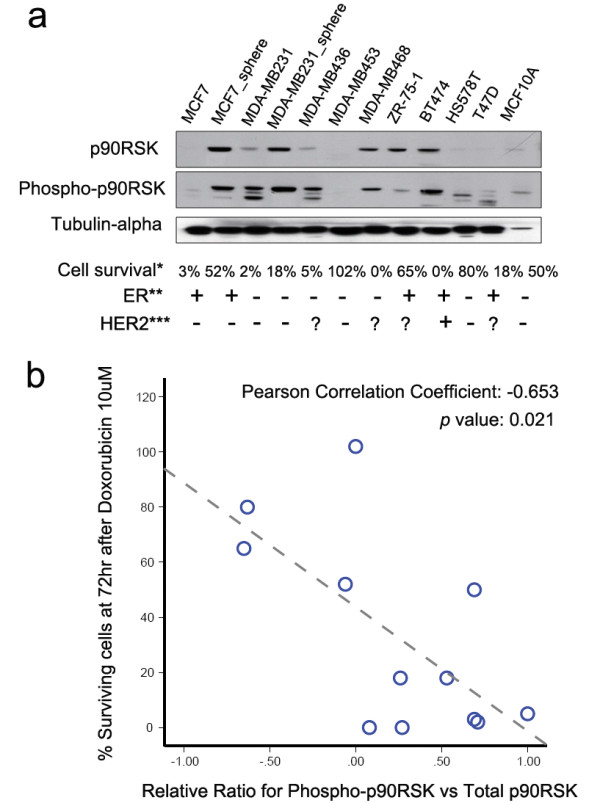
**The expression of phospho-p90RSK in various breast cancer cell lines and doxorubicin sensitivity.** The levels of protein expression of phospho-p90RSK and total p90RSK were examined in 11 breast cancer cell lines (**a**). The scattered plot for correlation analysis between relative phospho-p90RSK expression and sensitivity to doxorubicin is shown (**b**). Cell survival* denotes for the proportion of cancer cells surviving after doxorubicin 10uM treatment. The expression status of ER and HER2 in various breast cancer cell lines was determined by the works of **Subik et al. [14] and ***Neve et al. [15].

**Figure 2:**
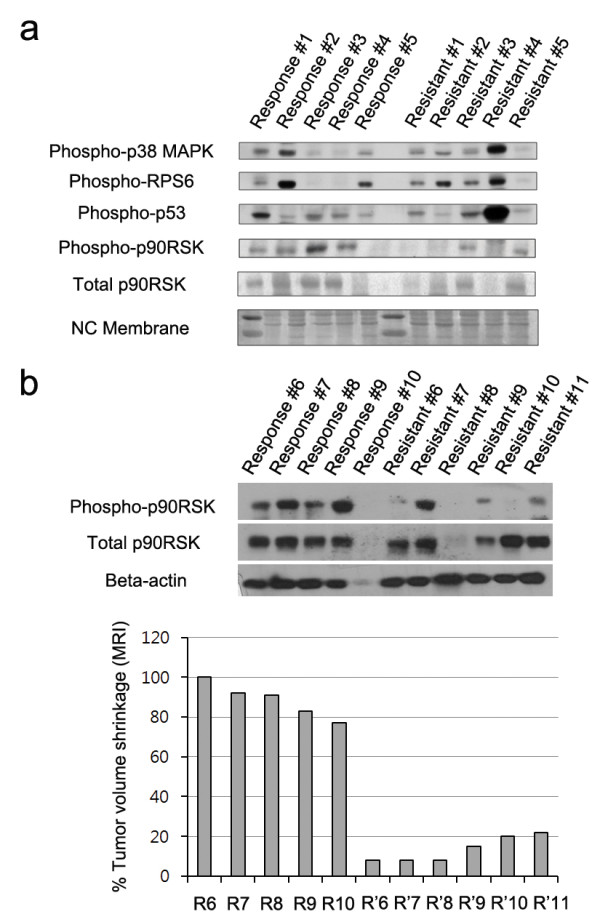
**The expression of phospho-p90RSK in human breast cancer undergoing neoadjuvant chemotherapy.** In 10 locally breast cancer patients, phospho-p90RSK and total p90RSK expression were measured by western blotting in core needle biopsy specimens of breast cancer before initiation of neoadjuvant chemotherapy. Patients were classified according to RECIST criteria determined by the treating physician (**a** and **b**). In 11 patients in whom the pre-chemotherapy and post-chemotherapy magnetic resonance imaging were available, the expression of phospho-p90RSK in tumor tissue and their corresponding radiologic tumor volume shrinkage are shown in (**b**).

We screened phospho-p90RSK expression level and the proportion of surviving cells after doxorubicin treatment in various breast cancer cell lines (Figure 1a). Relative phospho-p90RSK expression in these 12 breast cancer cells were inversely associated with the sensitivity to doxorubicin (Pearson correlation coefficient = −0.653, Figure 1b). The absolute expression level of phosphor-p90RSK showed similar association with the degree of response to doxorubicin but with borderline statistical significance (*p* = 0.083, Additional file 1: Figure S1). Then, we examined the phospho-p90RSK expression in fresh frozen tissue samples of 21 breast cancer before the initiation of neoadjuvant chemotherapy. When the patients were classified according to the RECIST criteria, patients who experienced clinical response to neoadjuvant chemotherapy had tumors with higher phospho-p90RSK expression (Mann–Whitney test *p* = 0.024 for phosphor-p90RSK, *p* = 0.004 for phosphor-p90RKS/total p90RSK, Figure 2a). Among the 21 patients, pre-chemotherapy and post-chemotherapy magnetic resonance imaging data (MRI) were available in 11 patients. The degree MRI-measured radiologic tumor shrinkage was higher in patients with high phospho-p90RSK expressing tumors (Figure 2b).

**Figure 3:**
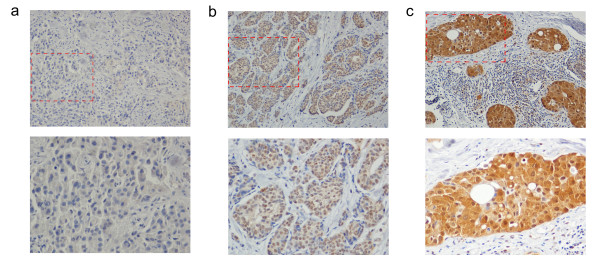
**Immunohistochemical staining against phospho-p90RSK in human breast cancer tissue.** Protein expression of phospho-p90RSK in human breast cancer tissue was examined by immunohistochemistry. In upper panels, the representative immunohistochemical staining images of phospho-p90RSK negative (**a**), weak (**b**), and strong (**c**) expression are shown (magnification X10). In lower panels, X20 magnified images corresponding to the red-dashed square are shown.

**Figure 4:**
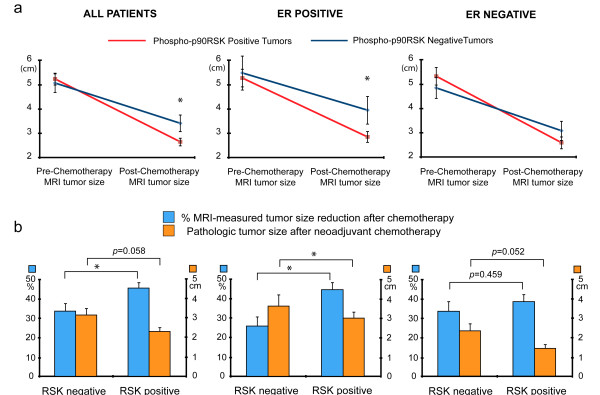
**Phospho-p90RSK expression and response to neoadjuvant chemotherapy.** In 112 locally advanced breast cancer patients, the expression of phospho-p90RSK expression and the radiologic tumor size shrinkage were examined. Pre-chemotherapy radiologic tumor size and postchemotherapy tumor size (**a**), proportional tumor size reduction, and post-chemotherapy pathologic tumor size (**b**) were examined in all tumors (left panel), estrogen receptor-positive tumors (middle panel), and estrogen receptor-negative tumors (right panel). * denotes for p < 0.05 from Student’s t-test.

We extended the clinical implication of phospho-p90RSK expression in pre-chemotherapy core needle biopsy specimens of 112 locally advanced breast cancer patients who underwent neoadjuvant chemotherapy with anthracyclin and taxane-based regimens. We chose to measure the expression level of phosphor-p90RSK expression by immunohistochemistry since measuring the relative phosphorylation ratio seemed impractical considering the semi-quantitative nature of the immunohistochemistry. The representative figures of immunohistochemical staining are shown in Figure 3. Among the 112 patients, 77 patients (70.5%) showed positive phospho-p90RSK expression. The association between phospho-p90RSK expression and various clinicopathologic tumor characteristics are shown in Table 1. Positive phospho-p90RSK expression was associated with younger age at diagnosis (p = 0.004). However, phospho-p90RSK did not show significant relationship with factors reported to affect the tumor response to neoadjuvant chemotherapy such as initial clinical stage or ER expression status [13]. Figure 4 shows the response to neoadjuvant chemotherapy according to the phospho-p90RSK expression. The pathologic extent of residual breast cancer after neoadjuvant chemotherapy was smaller in phospho-p90RSK positive tumors with borderline statistical significance. Furthermore, phospho-p90RSK positive tumors showed significant better response to neoadjuvant chemotherapy in terms of radiologic residual tumor extent and proportional tumor size reduction (Figure 4a and Figure 4b). The predictive value of phospho-p90RSK expression was more evident in ER-positive tumors when compared to ER-negative tumors. However, the phosphor-p90RSK expression did not show a statistically significant relationship with the incidence of pCR in both univariate and multivariate analysis (Additional file 2: Table S1). Only a borderline statistical significance was seen in multivariate regression analysis.

**Table 1:** Comparison of clinical and pathologic characteristics of breast cancer patients according to the phospho-p90RSK expression

		**Low P90RSK**	**High P90RSK**	
Mean age		55.3 (±10.4)	48.3 (±11.9)	0.004
Initial tumor size (cm)		5.1 (±2.3)	5.2 (2.4)	0.734
cT stage	cT1-2	18 (55%)	38 (48%)	0.339
	cT3-4	15 (45%)	41 (52%)	
cN stage	cN0-1	21 (64%)	40 (51%)	0.149
	cN2-3	12 (36%)	39 (49%)	
pCR	pCR no	29 (88%)	72 (91%)	0.414
	pCR yes	4 (12%)	7 (9%)	
Estrogen receptor	ER negative	18 (55%)	42 (53%)	0.530
	ER positive	15 (45%)	37 (47%)	
Progesterone receptor	PR negative	10 (67%)	36 (64%)	0.560
	PR positive	5 (33%)	20 (36%)	
HER2 overexpression	HER2 negative	10 (67%)	39 (70%)	0.527
	HER2 positive	5 (33%)	17 (30%)	

**Figure 5:**
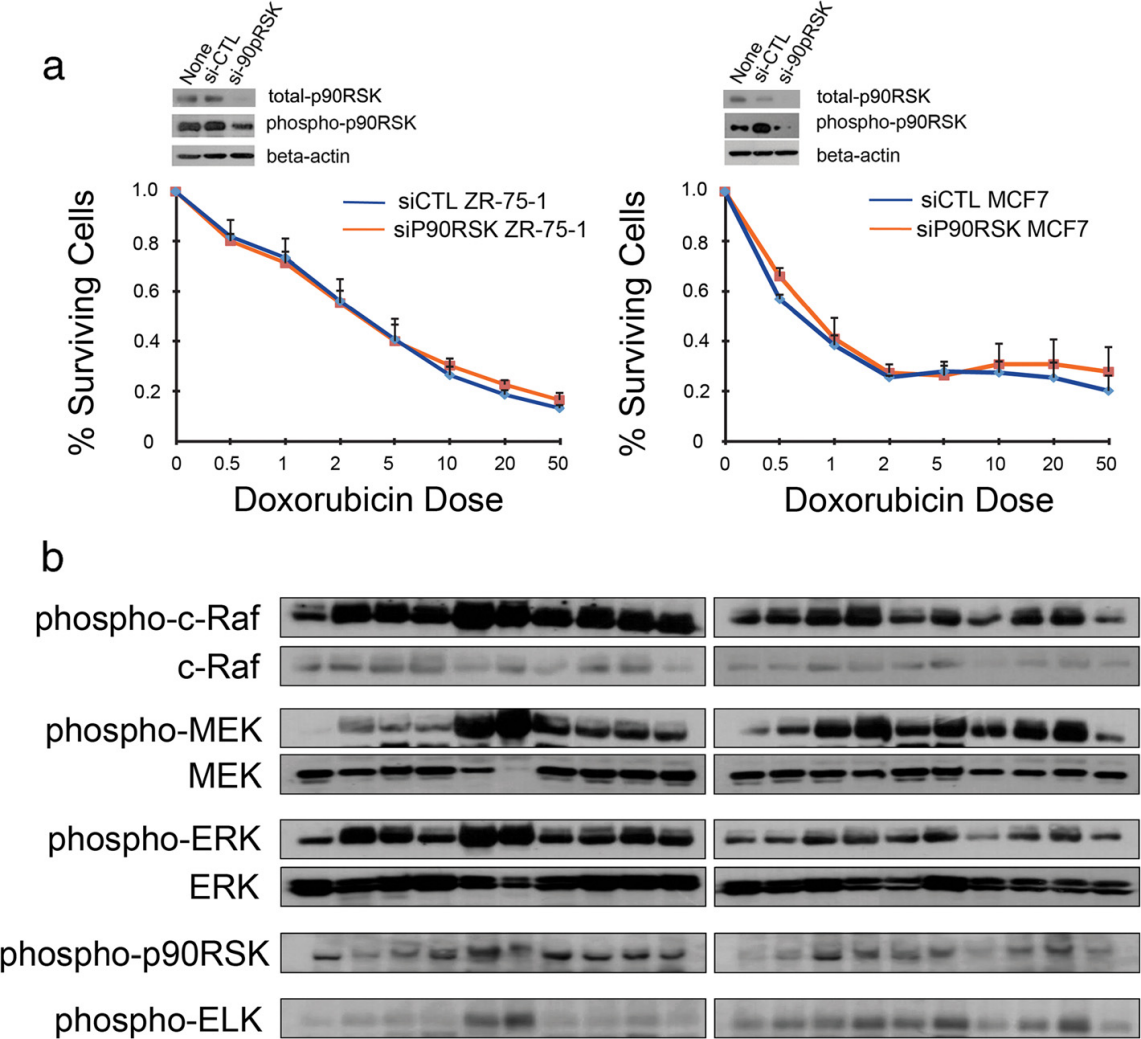
**Results of p90RSK gene silencing in cancer cell lines and various Raf/Ras/ERK pathway activation in human breast cancer.** p90RSK inhibition was done in MCF7 and ZR-75-1 cells by using siRNA against p90RSK. Student t-test showed no significant difference in cell proliferation in cells treated with siRNA against p90RSK and cells treated with scramble siRNA (**a**). The expression levels of total and phosphorylated various Raf/Ras/ERK pathway molecules were measured in primary breast cancer tissues from 20 breast cancer patients.

We next evaluated the functional importance of p90RSK in modulating chemotherapy response by silencing p90RSK with siRNA. We chose 2 ER-expressing breast cancer cell lines since the association between phosphor-p90RSK and chemotherapy responsiveness was significant in ER-positive tumors. Unlike the hypothesis, p90RSK silencing did not result result in decreased sensitivity to doxorubicin in ZR-75-1 and MCF7 breast cancer cell lines (Figure 5a). The expression of phospho-p90RSK was investigated in the context of Raf/MEK/ERK/p90RSK pathway activation in 20 primary breast cancer patients. In breast cancer tissues, the phospho-p90RSK expression was highly correlated with phospho-c-Raf (*p* = 0.012), phospho-MEK (*p* = 0.003), phospho-ERK (*p* = 0.009), and its downstream molecule phospho-ELK (p = 0.079), suggesting that the expression of phospho-p90RSK may reflect the whole Raf/MEK/ERK pathway and thereby mediating chemotherapy response.

## Discussion

In this study, we show that the degree of phosphorylation at p90RSK, a downstream molecule of ERK, is associated with the response to doxorubicin and taxane-based chemotherapy in breast cancer. By examining 12 breast cancer cell lines, we observed a significant relationship between the degree of phospho-p90RSK expression and survival after exposure to doxorubicin. Additionally, the expression of phospho-p90RSK measured by western blotting and immunohistochemistry in human breast cancer tissue was associated with the response to neoadjuvant chemotherapy in locally advanced breast cancer. Our results suggest the potential usefulness of measuring phospho-p90RSK as a predictive marker for response before the neoadjuvant chemotherapy.

The biologic role of p90RSK in cancer development and progression has recently been investigated in various types of malignancies. p90RSK is required in mTORC1 activation in BRAF-mutated melanoma cells which leads to increased growth in vitro [19]. p90RSK is also involved in invadopodia formation for cancer cell migration through the extracellular matrix [20]. Furthermore, it has been recently suggested that p90RSK is an important mediator of epithelial mesenchymal transition and cancer cell migration [21]. Based on these recent observations, p90RSK is now considered to be a potentially promising target for certain types of tumors [22].

In breast cancer, gene silencing p90RSK resulted in decreased number of tumor initiating cell phenotype represented by changes in surface marker such as CD44 and decreased ability to form mammosphere [23]. Additionally, Xian et al. [24] have shown that treatment with small molecules or small interfering RNA against p90RSK can induce cell death in FGFR1-mediated transformed cells. Our results showing the potential role of phospho-p90RSK as a predictive marker of chemotherapy response extend our understanding of the roles of p90RSK in breast cancer. Although a recent study suggested that the effect of p90RSK-induced cell proliferation can be modulated independently of ERK activation, our results show that the integrity of Ras/Raf/ERK and p90RSK pathway is well-maintained in human breast cancer tissues. Furthermore, gene silencing using siRNA against p90RSK did not affect the cancer cells’ sensitivity to doxorubicin suggesting the predictive role of p90RSK is the result of Ras/Raf/ERK/p90RSK pathway activity. Our results indicate that phospho-p90RSK can be a useful marker for predicting chemotherapy response but it may not be a suitable therapeutic target for functional modulation.

While the relationship between ERK signaling pathway in endocrine resistance is well-known in breast cancer [10], the role of this pathway including p90RSK in modulating chemotherapy response is yet to be explored. As mentioned before, exposure to doxorubicin in breast cancer cell lines resulted in phosphorylation of p90RSK which peaked 6 hours after the exposure [6]. Furthermore, MKP which dephosphorylate ERK1/2 and p38 MAPK inhibit the chemotherapy-induced JNK-related apoptotic pathway and contribute to the chemotherapy resistance [7]. Small et al. [25] have shown that transient or stable overexpression of MKP-1 reduced doxorubicin- or paclitaxel-induced apoptosis in MDA MB231 cells. However, there is also a contradictory report showing the lack of association between Ras/Raf/ERK pathway activation measured by immunohistochemistry and clinical benefit from chemotherapy when tumors of patients who participated in clinical trials were analyzed [4]. Our results show that tumors with increased phospho-p90RSK expression had 12% absolute benefit in terms of proportional size reduction during the neoadjuvant chemotherapy as measured by magnetic resonance imaging. Indeed, increased ERK pathway signaling was associated with enhanced apoptosis after anthracycline treatment in a neuroblastoma cell line [26].

Interestingly, our results show that the association between the chemotherapy response and the degree of p90RSK phosphorylation is more evident in ER positive tumors. Although the underlying mechanism is unknown, it is possible to speculate that phospho-p90RSK can increase the transcriptional activity of AF-1 of ER by phosphorylating Ser (167) [27]. In accordance with out results, the phosphorylation of ER Ser(167) has been shown to be correlated with phospho-p90RSK expression and was associated with better treatment outcome in ER positive breast cancer patients [28,29]. However, the relationship between phospho-p90RSK and treatment outcome in breast cancer should further be explored in a larger cohort of patients since a recent study showed that the p90RSK mRNA level was higher in triple negative breast cancer and was associated with poor survival [23].

Our study carries some limitations. First we could not eliminate the possibility of selection bias since our study is a retrospective study including relatively small number of patients who underwent neoadjuvant chemotherapy. Second, the predictive role of phospho-p90RSK should be independently addressed in patients who receive adjuvant chemotherapy since the response to neoadjuvant chemotherapy and outcome after adjuvant chemotherapy may differ [30]. Especially, we could not find a statistically significant relationship between phosphor-p90RSK expression and the incidence of pCR after neoadjuvant chemotherapy which is a well-known prognostic factor. Only a borderline significance was seen in multivariate regression analysis between phosphor-p90RSK and pCR. One possible explanation would be that, in our data, the relationship between the phosphor-p90RSK expression and chemotherapy response was significant only in ER positive tumors. ER positive tumors show significantly lower incidence of pCR when compared to ER negative tumors and the both tumors also differ in chemotherapy response patterns [31]. However, it is still important to predict chemotherapy-responsiveness in terms of selecting patients who will become candidates for successful breast conservation regardless of the likelihood of achieving pCR. Additionally, we were not able to apply other pathologic response parameters such as residual cancer burden (RCB) index as proposed by Symmans et al. [32]. Finally, the effector molecule which modulates the relationship between the Ras/Raf/ERK/p90RSK pathway activity and the chemotherapy sensitivity should be investigated in future studies. Our data on the association of phosphor-p90RSK and chemotherapy sensitivity can be the results from different ERK activity and proliferation activity in each cell lines.

## Conclusion

In conclusion, by using human breast cancer samples and cancer cell lines, we show that phospho-p90RSK can be a potential marker for chemotherapy response in ER positive breast cancer patients. The prognostic role of phospho-p90RSK in breast cancer patients as well as the functional mechanism underlying the association between Ras/Raf/ERK/p90RSK pathway activity and chemotherapy response should further be explored.

## Abbreviations

ER: Estrogen receptor; ERK: Extracellular signal-regulated kinases; HER2: Human epidermal growth factor receptor 2; MEK: Mitogen-activated protein kinase/extracellular signal-regulated kinase kinase; MKP: Mitogen-activated protein kinase phosphatase; MTORC1: mTOR Complex 1; NSABP: National Surgical Adjuvant Breast and Bowel Project; P90RSK: p90 ribosomal S6 kinase; PR: Progesterone receptor; RECIST: Response Evaluation Criteria in Solid Tumors; siRNA: Small interfering RNA.

## Competing interests

The authors declare no competing interests.

## Authors’ contributions

DYN, HGM and JKY designed the study. JKY, HYL, and KML conducted western blot and cell line studies. HGM, SA, HCS, WH collected and analyzed the clinical data. WH and DYN provided the patients’ tissue and paraffin blocks. HSK, MY, and HGM conducted immunohistochemistry and interpreted the results. JJ and IS performed immunoblots for primary breast cancer tissues. All authors read and approved the final manuscript.

## Pre-publication history

The pre-publication history for this paper can be accessed here:

http://www.biomedcentral.com/1471-2407/12/585/prepub

## Supplementary Material

Additional file 1 **Figure S1.** Scatter plot for the relationship between phosphor-p90RSK expression and chemotherapy sensitivity in 12 breast cancer cell lines. Cell survival denotes for the proportion of cancer cells surviving after doxorubicin 10uM treatment. Pearson correlation coefficient and p value were derived from correlation analysis. Click here for file

Additional file 2 **Table S1.** Univariate and Multivariate analysis for factors affecting pCR. Click here for file
