# Supplementary material for: Phosphorylation of p90RSK is associated with increased response to neoadjuvant chemotherapy in ER-positive breast cancer
Source: BMC Cancer. 2012 Dec 10;12:585. doi: 10.1186/1471-2407-12-585 (PMC3523086; doi:10.1186/1471-2407-12-585)
Supplement: Additional file 1 — Figure S1. Scatter plot for the relationship between phosphor-p90RSK expression and chemotherapy sensitivity in 12 breast cancer cell lines. Cell survival denotes for the proportion of cancer cells surviving after doxorubicin 10uM treatment. Pearson correlation coefficient and p value were derived from correlation analysis. [file 1471-2407-12-585-S1.doc]

Figure S1 Scatter plot for the relationship between phosphor-p90RSK expression and chemotherapy sensitivity in 12 breast cancer cell lines. Cell survival denotes for the proportion of cancer cells surviving after doxorubicin 10uM treatment. Pearson correlation coefficient and p value were derived from correlation analysis
