# Supplementary material for: Phosphorylation of p90RSK is associated with increased response to neoadjuvant chemotherapy in ER-positive breast cancer
Source: BMC Cancer. 2012 Dec 10;12:585. doi: 10.1186/1471-2407-12-585 (PMC3523086; doi:10.1186/1471-2407-12-585)
Supplement: Additional file 2 — Table S1. Univariate and Multivariate analysis for factors affecting pCR. [file 1471-2407-12-585-S2.doc]

Table S1 **Univariate and Multivariate analysis for factors affecting pCR**

| Univariate analysis |  |  |  |  |
| --- | --- | --- | --- | --- |
|  | pCR no | pCR yes | p |  |
| Initial tumor size (MRI) | 5.5 (±2.35) | 4.1 (±2.03) | 0.011 |  |
| Post-chemotherapy tumor size (MRI) | 1.9 (±1.54) | 3.2 (±1.77) | 0.003 |  |
| MRI tumor reduction (%) | 57.6 (±28.6) | 38.6 (±25.3) | 0.003 |  |
| Mean age | 50.5 (±12.7) | 49.8 (±7.8) | 0.816 |  |
| p90RSK | p90RSK low | 29 (28.7%) | 4 (36.4%) | 0.414 |
|  | p90RSK high | 72 (71.3%) | 7 (63.6%) |  |
| cT stage | cT1-T2 | 46 (45.5%) | 10 (90.9%) | 0.008 |
|  | cT3-cT4 | 55 (54.5%) | 1 (9.1%) |  |
| cN stage | cN0-1 | 54 (53.5%) | 7 (63.6%) | 0.751 |
|  | cN2-3 | 47 (46.5%) | 4 (36.4%) |  |
| Estrogen receptor | ER negative | 52 (51.5%) | 8 (72.7%) | 0.217 |
|  | ER positive | 49 (48.5%) | 3 (27.3%) |  |
| HER2 | HER2 negative | 44 (71.0%) | 5 (55.6%) | 0.444 |
|  | HER2 positive | 18 (29.0%) | 4 (44.4%) |  |
| Multivariate analysis |  |  |  |  |
| Factor | p | HR | 90% C.I. for HR |  |
| Age | .296 | .948 | .858 | 1.048 |
| Pre-chemotherapy tumor size | .017 | .317 | .123 | .817 |
| cT3-T4 (vs cT1-T2) | .630 | .446 | .017 | 11.952 |
| cN2-N3 (vs cN0-N1) | .632 | .560 | .052 | 6.005 |
| ER negative (vs ER positive) | .453 | 2.136 | .294 | 15.531 |
| HER2 positive (vs HER2 negative) | .081 | .146 | .017 | 1.272 |
| p90RSK high (vs p90RSK low) | .089 | 7.422 | .738 | 74.631 |

For univariate analysis, p values were calculated from Student’s t-test and chi-square test for continuous and categorical variables, respectively. For multivariate analysis, multiple regression analysis was used. Age and tumor size were entered into the model as continuous variable
